# A network meta-analysis of endocrine adverse events induced by immune checkpoint inhibitors in colorectal cancer

**DOI:** 10.3389/fimmu.2026.1798732

**Published:** 2026-07-10

**Authors:** Boyu Chen, Jing Liu, Kexin Gan, Liqun Yang, Peng Qiu, Boqing Ma, Wen Chen

**Affiliations:** 1 Graduate School, Hebei North University, Zhangjiakou, Hebei, China; 2 Department of Endocrinology, Hebei General Hospital, Shijiazhuang, Hebei, China; 3 Hebei Key Laboratory of Metabolic Diseases, Shijiazhuang, Hebei, China; 4 Department of Laboratory Medicine (Clinical Laboratory), Hebei General Hospital, Shijiazhuang, Hebei, China; 5 Department of Anus and Intestine Surgery, Shijiazhuang People's Hospital, Shijiazhuang, China

**Keywords:** colorectal cancer, combination therapy, endocrine adverse events, immune checkpoint inhibitors, network meta-analysis

## Abstract

Immune checkpoint inhibitor (ICI) therapy for colorectal cancer (CRC) can be accompanied by endocrine adverse events, yet the comparative risk across commonly used regimens remains unclear. We therefore conducted a network meta-analysis of randomized controlled trials in CRC published up to November 22, 2025, estimating risk ratios (RRs) with 95% confidence intervals (CIs) and assessing risk of bias. Six RCTs were included. Relative to conventional therapy, ICI-based regimens were associated with a higher thyroid-related toxicity burden. Pembrolizumab and ICI+tyrosine kinase inhibitor (TKI) significantly increased the risk of hypothyroidism, whereas hyperthyroidism was significantly higher with ICI+TKI and ICI plus chemotherapy plus an anti-angiogenic antibody (ICI+Chem+Antiangio-Ab). Grade 1–2 adverse events were consistently increased across ICI-based treatments. For thyroiditis, diabetes mellitus, adrenal insufficiency, and grade 3–4 adverse events, effect estimates were imprecise with wide 95% CIs; nevertheless, SUCRA rankings tended to place ICI+TKI toward the higher-risk end for thyroiditis and diabetes. These findings indicate that ICI-containing strategies in CRC increase risks of endocrine adverse events—particularly for thyroid dysfunction—supporting the need for proactive endocrine monitoring and standardized management, while highlighting the limited precision of current evidence for rarer endpoints and severe toxicity.

**Systematic Review Registration:**

https://www.crd.york.ac.uk/PROSPERO/view/CRD42023469312, identifier CRD42023469312.

## 1 Introduction

Colorectal cancer (CRC) remains a significant global health challenge, ranking third in incidence and second in cancer-related mortality worldwide. Global estimates in 2020 reported roughly 1.9 million new CRC cases and about 0.93 million deaths (1). Despite progress in screening and multimodal therapy, outcomes for advanced CRC remain disappointing, with low five-year survival rates and ongoing clinical challenges (2). Thus, improving survival in advanced CRC remains a major focus in oncology. In recent years, cancer immunotherapy has opened new therapeutic avenues for CRC, and immune checkpoint inhibitors (ICIs) in particular have generated considerable interest. However, while ICIs can provide durable antitumor effects, they also bring distinct immune-related toxicities, making safety management an essential component of clinical care (3).

ICIs reinvigorate antitumor immunity by blocking inhibitory checkpoints between tumor cells and immune cells (for example, PD-1/PD-L1 or CTLA-4), thereby lifting the brakes on T cells and restoring their ability to target tumors. In CRC, the clinical benefit of ICIs is largely confined to the subgroup of patients with high microsatellite instability or mismatch repair deficiency (MSI-H/dMMR), which represents roughly 5% of advanced CRC cases (4). Multiple clinical trials have demonstrated that PD-1 inhibitors (such as pembrolizumab and nivolumab), with or without CTLA-4 blockade (ipilimumab), can induce durable responses and significantly improve outcomes in MSI-H/dMMR metastatic CRC (5, 6). Notably, the KEYNOTE-177 trial showed that first-line pembrolizumab significantly prolonged progression-free survival and improved response rates compared to chemotherapy (5). Based on these data, immunotherapy has become a key standard-of-care option for MSI-H/dMMR metastatic CRC, reshaping previous treatment paradigms (4).

Alongside these efficacy gains, the toxicity profile of ICI therapy has shifted. Immune-related adverse events (irAEs) arise from excessive immune activation and can affect multiple organ systems, most commonly the skin, gastrointestinal tract, liver, and endocrine glands (7). Endocrine irAEs deserve particular attention because they result from loss of immune tolerance, with autoreactive immune cells attacking endocrine glands. Clinically, endocrine toxicities include various glandular dysfunctions: for example, thyroiditis (a brief hyperthyroid phase followed by hypothyroidism), hypothalamic-pituitary inflammation (secondary hypopituitarism), adrenal insufficiency, and insulin-dependent diabetes mellitus (7, 8). Endocrine irAEs generally occur early in the treatment course: most events cluster within the first six months after initiating ICIs (7). For instance, thyroiditis typically produces a transient thyrotoxic phase around eight weeks into therapy, with hypothyroidism often developing about 6–14 weeks later. Hypophysitis usually emerges in the range of 9–26 weeks. Likewise, primary adrenalitis and ICI-associated type 1 diabetes most often appear within weeks to months of therapy initiation, although later-onset cases do occur (9). Therefore, endocrine function monitoring should be intensified during the first few months of ICI therapy to enable early detection of abnormalities (7). About 10% of patients receiving ICIs may develop some form of endocrine dysfunction, and a portion of these patients will require long-term hormone replacement therapy. Importantly, unlike many other irAEs, endocrine adverse events often tend to be irreversible (for example, evolving into permanent hypothyroidism or persistent pituitary axis failure), necessitating long-term follow-up and management by endocrinology specialists (7, 8). These complications not only increase the complexity of clinical care but also can disrupt treatment continuity and impair patients’ quality of life. Consequently, early recognition and standardized management of endocrine irAEs have been repeatedly emphasized in reviews and clinical guidelines.

Despite a growing literature on ICI safety, systematic comparative evidence focusing specifically on endocrine adverse events in CRC remains limited. In fact, most available safety data come from melanoma, lung cancer, and other malignancies, or are reported only in aggregate form (9). For CRC, clear conclusions are still lacking on how endocrine risk varies among different immunotherapy strategies — for example, PD-1 monotherapy, PD-1 plus CTLA-4 blockade, or other combination approaches — and on the relative ranking of these regimens. This evidence gap makes it challenging to weigh toxicity risks alongside therapeutic benefits for individualized treatment decisions. Network meta-analysis, which integrates direct and indirect comparisons across multiple regimens, could provide a higher level of evidence for estimating relative risks and generating treatment rankings (9). However, in CRC specifically, network meta-analyses examining immunotherapy-related endocrine toxicity remain scarce, underscoring the need for systematic comparative research.

In light of these gaps, this study will employ a network meta-analysis to systematically evaluate the relative risk of endocrine adverse events and differences in severity across various ICI-based treatment regimens in CRC patients. We will synthesize evidence from published randomized controlled trials (RCTs) to compare multiple immunotherapy strategies — including different ICI monotherapies and combination regimens — characterize the full spectrum of endocrine toxicity, and rank the associated risks. We hypothesize that endocrine toxicity profiles will differ depending on the regimen, and that certain combination therapies may be associated with higher risks of specific endocrine events (9). By integrating both direct and indirect evidence to generate risk rankings, this analysis aims to provide a more structured evidence base to inform regimen selection and endocrine monitoring strategies, ultimately supporting an optimal balance between efficacy and safety in clinical practice.

## 2 Materials and methods

### 2.1 Registration

Our network meta-analysis (NMA) adhered to the Preferred Reporting Items for Systematic Reviews and Meta-Analyses (PRISMA) 2020 statement when applying for prospective registration in the International Prospective Register of Systematic Reviews (PROSPERO; ID: CRD42023469312) (**Supplementary S1**).

### 2.2 Search strategy

The literature search was conducted by the authors and an additional independent investigator. The databases searched included PubMed, Embase, and the Cochrane Library. The search terms comprised “PD-L1 Inhibitors”, “Tremelimumab”, “Pembrolizumab”, “Nivolumab”, “Cemiplimab”, “Sintilimab”, “Toripalimab”, “Tislelizumab”, “Camrelizumab”, “Atezolizumab”, “Durvalumab”, “Avelumab”, “CTLA-4”, “PD-1”, “PD-L1”, and “immune checkpoint inhibitor”. The search terms within the same category were combined using “OR”, while those from different categories were combined with “AND”. Subject words were combined with free-text terms for the search. Only English-language articles published before November 22, 2025, were eligible. The full electronic search strategies (for all databases) are provided in **Supplementary S2**.

### 2.3 Study selection: inclusion and exclusion criteria

The inclusion and exclusion criteria were formulated according to the evidence-based PICO framework (Population, Intervention, Comparator, and Outcomes). Two reviewers independently screened all titles, abstracts, and full texts to determine eligibility; disagreements were resolved through discussion and consensus, with adjudication by a third reviewer when necessary.

The inclusion criteria were as follows (1): study design: RCTs; (2) population (P): patients with colorectal tumors; (3) intervention (I): ICI monotherapy or ICIs combined with conventional treatment regimens; (4) comparator (C): no restrictions on comparators (e.g., chemotherapy, targeted therapy, supportive care, or investigator’s choice of treatment); and (5) outcomes (O): reporting at least one endocrine-related adverse event/safety endpoint, including hyperthyroidism, hypothyroidism, thyroiditis, adrenal insufficiency, diabetes mellitus, and adverse event grades. Adverse events were graded according to the National Cancer Institute Common Terminology Criteria for Adverse Events, version 4.0.

The exclusion criteria were as follows: (1) non-randomized studies (e.g., single-arm studies, retrospective studies, case–control studies, case series, and case reports); (2) non-original publications, including reviews, systematic reviews/meta-analyses, commentaries, editorials/letters, correspondence, guidelines/consensus statements, conference abstracts, or proceedings; (3) animal experiments, *in vitro* studies, or studies not involving colorectal tumors; (4) studies in which the target outcome data could not be extracted or calculated (e.g., endocrine adverse event counts/denominators were not reported and remained unavailable after contacting the authors); and (5) duplicate publications or multiple reports from the same trial: when more than one article reported the same study, only the report with the most complete information and the most recent follow-up was included. Where necessary, complementary information across reports was consolidated, but the same outcome was not double-counted.

### 2.4 Data extraction

Data were extracted using a predesigned and pilot-tested standardized electronic data extraction form (Excel). Two reviewers independently extracted data and cross-checked the results; any discrepancies were resolved through discussion and consensus, with adjudication by a third reviewer when necessary. The following information was extracted from each included study: clinical trial registration number (NCT), first author and year of publication, study region/country, trial phase, participant characteristics (e.g., cancer type/molecular subtype and line of therapy), total sample size and sample size per arm, treatment regimens (detailed descriptions of interventions and comparators, including drugs, doses, and schedules), follow-up duration, and the grading criteria and reporting approach for endocrine adverse events. For outcome data, we extracted the number of events and the corresponding total sample size in each arm for the following endocrine adverse events: hyperthyroidism, hypothyroidism, thyroiditis, adrenal insufficiency, and diabetes mellitus. If multiple severity categories were reported (e.g., all-grade and grade ≥3), data for each category were extracted separately according to the prespecified analytical plan. For missing or unclear information, we prioritized verification using the main text, appendices/**Supplementary Materials**, and trial registry records.

### 2.5 Risk of bias assessment

The methodological quality of included studies was assessed using the revised Cochrane Risk of Bias tool (RoB 2) (10), covering five sources of biases: randomization process, deviations from intended interventions, missing outcome data, measurement of the outcome, and selection of the reported result.

### 2.6 Statistical analysis

Network meta-analysis was performed in a frequentist framework using Stata 18.0. All outcomes were binary and are reported as risk ratios (RRs) with 95% confidence intervals (95% CIs); two-sided P values <0.05 were considered statistically significant. We first constructed the evidence network and generated network plots. Under the consistency assumption, network meta-analysis models were fitted to produce league tables and the surface under the cumulative ranking curve (SUCRA), and mean ranks (MeanRank) were reported for treatment ranking. Because no closed loops were formed in the outcome-specific networks, we did not perform a global test of consistency, loop-specific inconsistency assessment, or local inconsistency testing based on the node-splitting approach. Publication bias/small-study effects were evaluated using comparison-adjusted funnel plots; given the limited number of included studies, this analysis was conducted only as an exploratory assessment for outcomes with a relatively sufficient number of studies.

## 3 Results

### 3.1 Study selection process and characteristics of included studies

**Figure 1:**
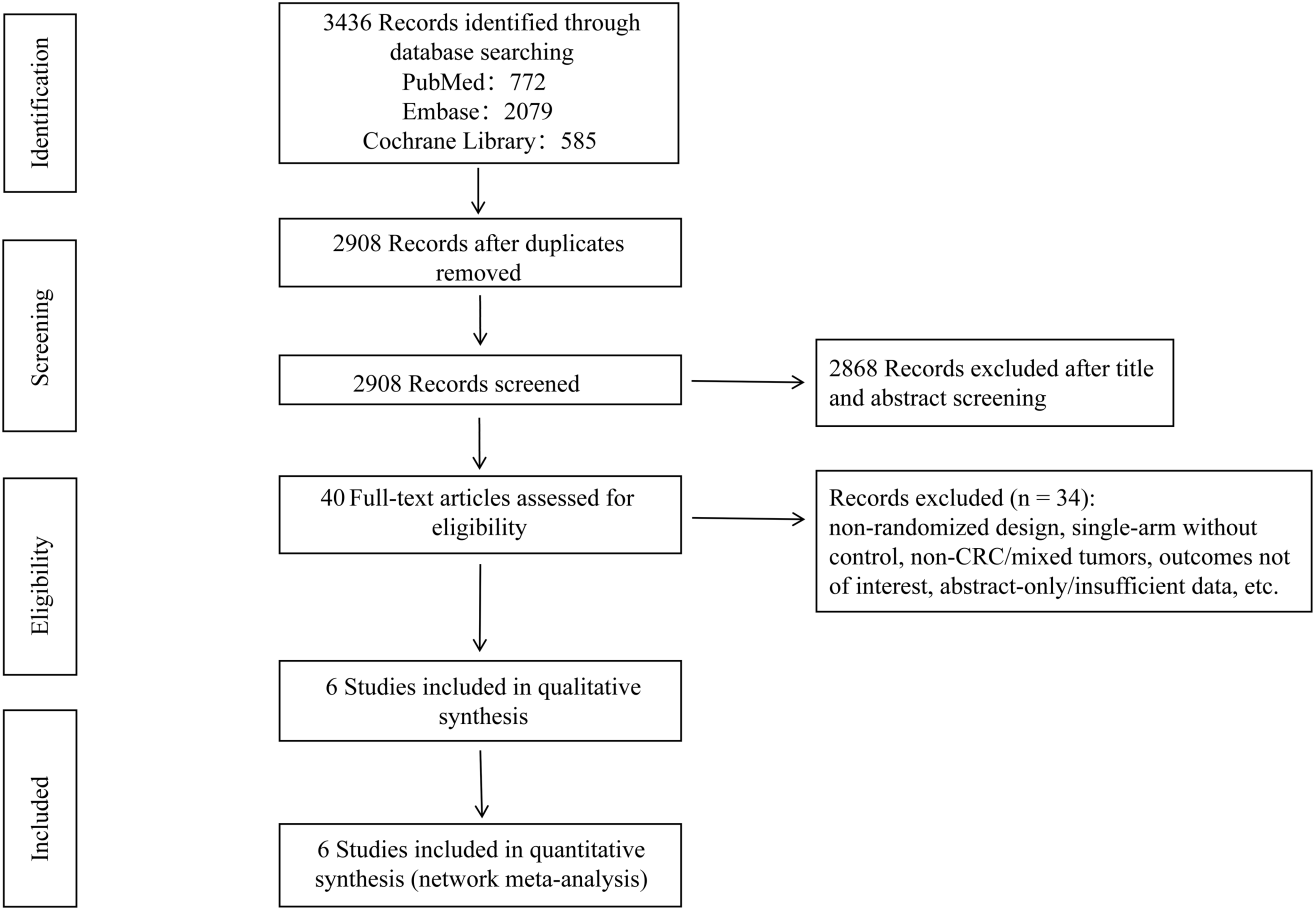
Literature search and study selection.

A total of 3,436 records were identified from electronic databases. Following duplicate removal, 2908 records remained. After screening titles and abstracts, 40 articles were retrieved for full-text review. Of these, 34 were further excluded, with reasons detailed in **Supplementary Table 4**. Ultimately, six RCTs (11–16) were included, comprising 1,648 patients (**Figure 1**). Among these, three studies were phase II trials and three were phase III trials. Baseline characteristics of the included trials are summarized in **Table 1**.

**Table 1:** Baseline characteristics of included randomized controlled trials.

Study (author, name, year, phase)	Clinical trial gov. no.	Publication type	Region/population	Median age (years)	Study period (enrollment/cutoff)	Total patients (male/female)	Treatment line number	Groups	No. patients	Median follow-up (months)
Taïeb et al, SAMCO-PRODIGE 54, 2023, II	NCT03186326	Full text	France	66	2018-04–24 to 2022-05-23	122 (57/65)	Second-line dMMR/MSI mCRC	Avelumab vs investigator’s choice chemotherapy ± targeted agents	61/61	33.3
Pan et al, XELOX+Bev ± PD1-T cells, 2024, III	NCT03950154	Full text	Chinese	59	2019-03–20 to 2022-09-09	202 (104/98)	First-line mCRC	XELOX+Bevacizumab+PD1-T vs XELOX+Bevacizumab	100/102	19.5
Mettu et al, BACCI, 2022, II	NCT02873195	Full text	Predominantly White	58	2017-09–25 to 2018-06-28	128 (51/77)	Refractory mCRC after ≥2–3 prior regimens	capecitabine bevacizumab with atezolizumab vs capecitabine bevacizumab with placebo	82/46	20.9
Tabernero et al, MODUL cohort 2, 2022, II	NCT02291289	Full text	International, mainly European	62	2015-04–17 to 2021-03-24	445 (297/148)	First-line mCRC	Fluoropyrimidine bevacizumab atezolizumab vs Fluoropyrimidine bevacizumab	297/148	20.3
André et al, KEYNOTE-177 final, 2025, III	NCT02563002	Full text	Western Europe/North America	63	2018–07 to 2023-07-17	307 (153/154)	First-line MSI-H/dMMR mCRC	Pembrolizumab vs FOLFOX6 or FOLFIRI	153/154	73.3
Kawazoe et al, LEAP-017, 2024, III	NCT04776148	Full text	Asia Western Europe/North America Rest of the world	58	2021-04–08 to 2021-12-21	480 (278/202)	Refractory pMMR/MSS mCRC after standard therapies	Lenvatinib + Pembrolizumab vs Regorafenib or FTD/TPI	241/239	18.6

### 3.2 Risk of bias assessment results

**Figure 2:**
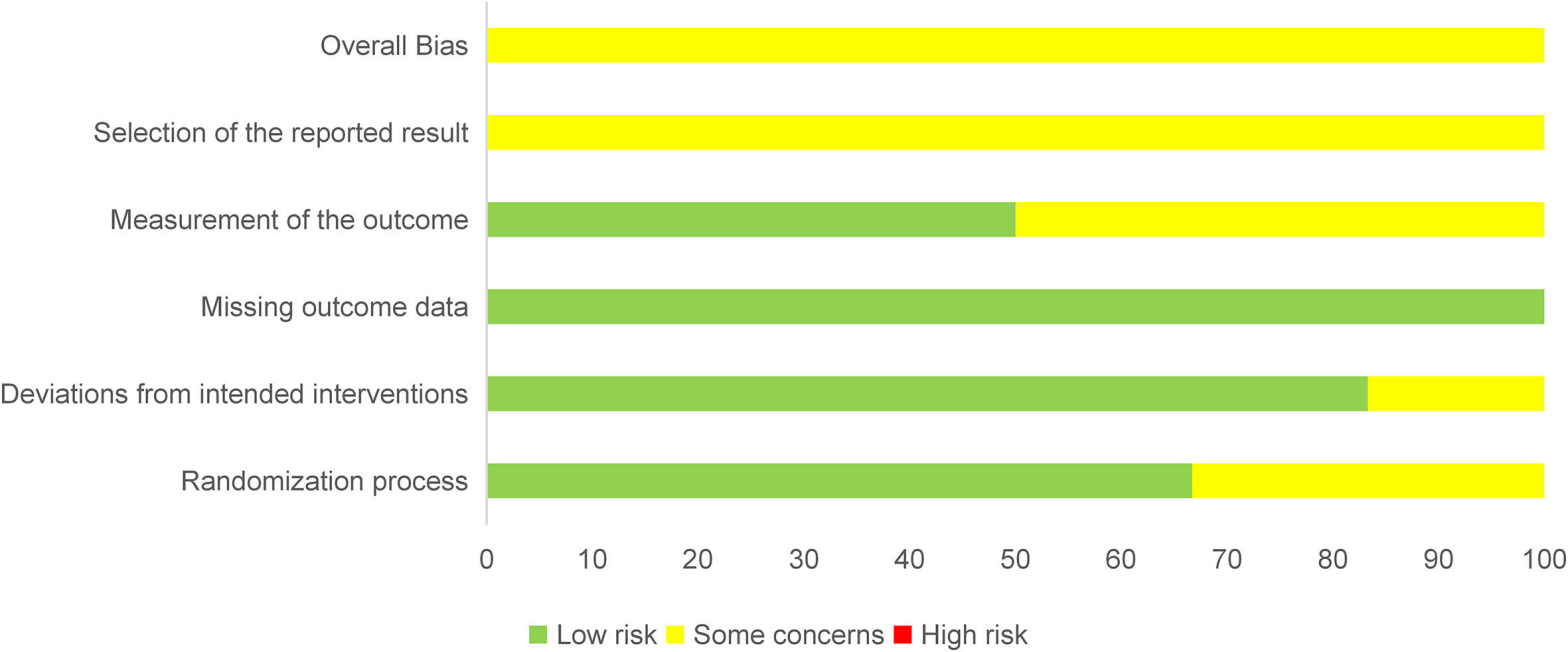
Overview of risk of bias in the included studies. Green represents low risk, yellow represents some concerns, and red represents high risk.

The RoB 2 assessment indicated that most of included studies were judged as low risk for the randomization process, deviations from intended interventions, missing outcome data; and outcome measurement. Notably, no assessment domain was judged as “high risk” (**Figure 2**). Overall, the methodological quality of the included studies was moderate.

### 3.3 Network meta-analysis results

#### 3.3.1 Network evidence plot

**Figure 3:**
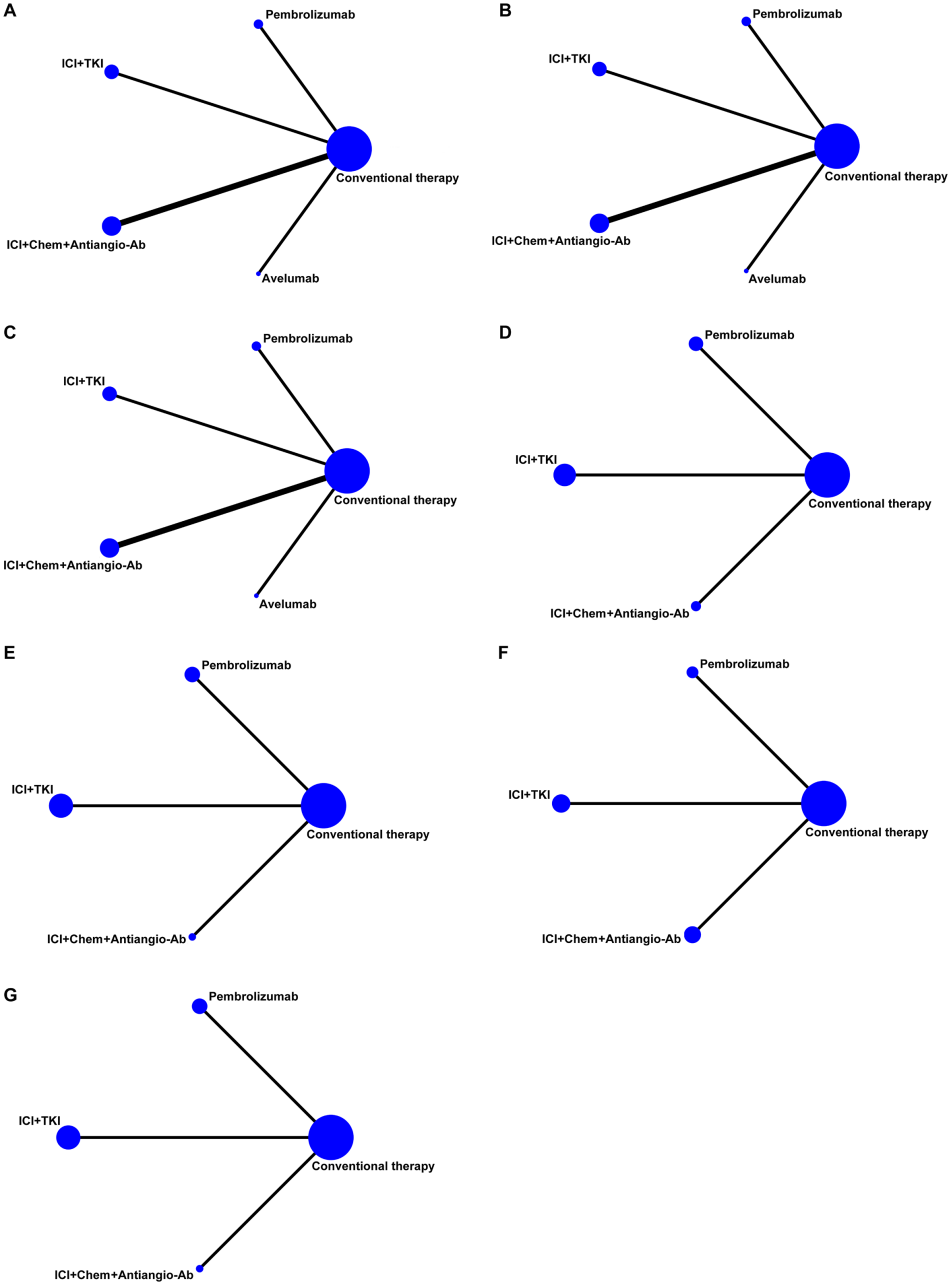
Network plots of different treatment regimens for hypothyroidism **(A)**, hyperthyroidism **(B)**, grade 1–2 adverse events **(C)**, adrenal insufficiency **(D)**, diabetes mellitus **(E)**, thyroiditis **(F)**, and grade 3–4 adverse events **(G)**. In the network plots, nodes represent interventions, with node size proportional to the cumulative sample size of each intervention; edges represent direct comparisons, with edge thickness proportional to the number of studies contributing to each comparison.

This network meta-analysis included five studies reporting hypothyroidism, five reporting hyperthyroidism, three reporting adrenal insufficiency, three reporting diabetes mellitus, three reporting thyroiditis, and six reporting adverse event grades. As shown in **Figures 3A–C**, five treatment regimens were evaluated in trials reporting hypothyroidism, hyperthyroidism, and grade 1–2 adverse events: conventional therapy, pembrolizumab, an ICI combined with a tyrosine kinase inhibitor (TKI) (ICI+TKI), an ICI combined with chemotherapy plus an anti-angiogenic antibody (ICI+Chem+Antiangio-Ab), and avelumab. As shown in **Figures 3D–G**, four treatment regimens were evaluated in trials reporting adrenal insufficiency, diabetes mellitus, thyroiditis, and grade 3–4 adverse events: conventional therapy, pembrolizumab, ICI+TKI, and ICI+Chem+Antiangio-Ab.

#### 3.3.2 League tables and cumulative ranking probabilities

**Figure 4:**
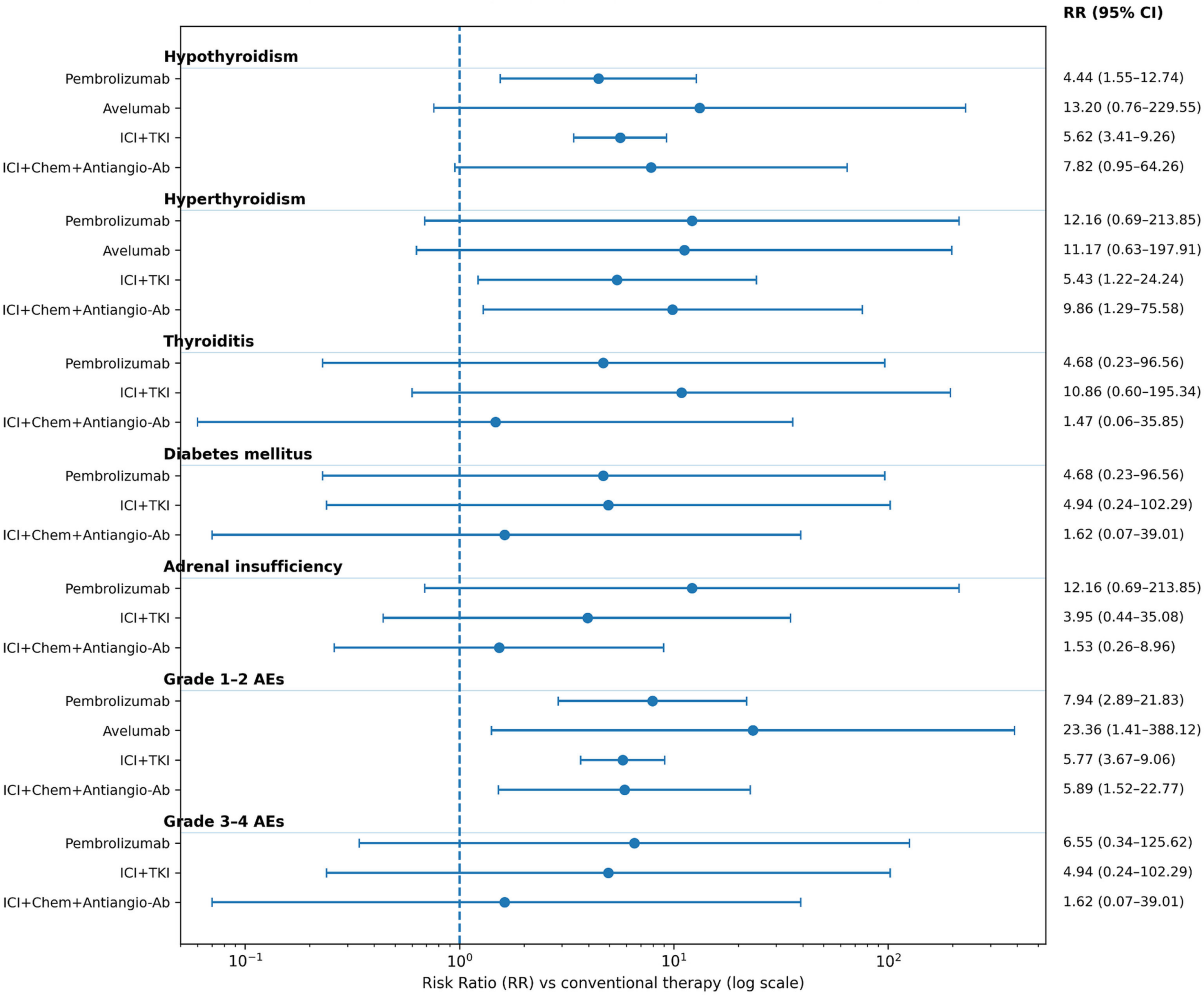
Summary of endocrine adverse events (RR vs conventional therapy). Network meta-analysis estimates are presented as risk ratios (RRs) with 95% confidence intervals (CIs) on a log scale; the dashed line indicates RR = 1. Points and horizontal lines denote RR estimates and 95% CIs for each regimen across endocrine endpoints and grade-stratified adverse events. Abbreviations: ICI, immune checkpoint inhibitor; TKI, tyrosine kinase inhibitor; Antiangio-Ab, anti-angiogenic antibody.

**Figure 5:**
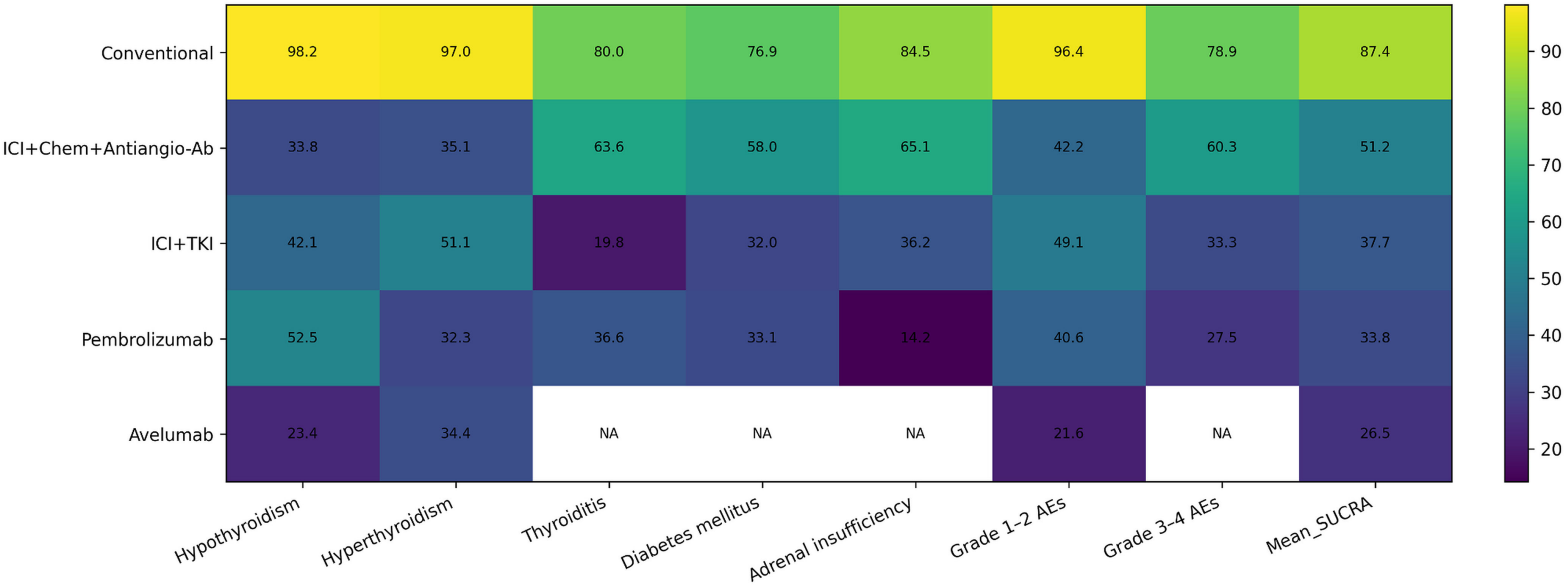
Multiple treatment comparison for endocrine adverse events based on SUCRA values. Heatmap shows SUCRA (%) for each regimen across endocrine adverse events; higher SUCRA indicates lower incidence of an endocrine adverse event. Mean_SUCRA denotes the average SUCRA across endpoints. NA indicates outcomes for which avelumab was not included in the network and SUCRA could not be estimated.

Detailed league tables (A) and cumulative ranking probability plots (B) for each endpoint are provided in **Supplementary Figure 1**-**S7**. Main findings are summarized in **Figure 4** (effect sizes vs conventional therapy) and **Figure 5** (SUCRA-based ranking summary).

**Figure 4** summarizes the network meta-analysis estimates of endocrine adverse events for different ICI-based regimens compared with conventional therapy. Overall, the endocrine toxicity profile of ICI-containing regimens shows an upward shift relative to conventional therapy. For hypothyroidism, pembrolizumab (RR = 4.44, 95% CI 1.55–12.74) and ICI+TKI (RR = 5.62, 95% CI 3.41–9.26) significantly increased risk versus conventional therapy. While point estimates were also elevated for avelumab (RR = 13.20, 95% CI 0.76–229.55) and ICI+Chem+Antiangio-Ab (RR = 7.82, 95% CI 0.95–64.26), these were not statistically significant. For hyperthyroidism, the risk elevation was more pronounced for combination strategies: both ICI+TKI (RR = 5.43, 95% CI 1.22–24.24) and ICI+Chem+Antiangio-Ab (RR = 9.86, 95% CI 1.29–75.58) indicated significantly increased risk compared with conventional therapy, collectively supporting a regimen-intensity gradient for thyroid toxicity. For relatively low-incidence endpoints, including thyroiditis, diabetes mellitus, and adrenal insufficiency, point estimates were generally RR>1, but non-significant due to wide 95% CIs. In grade-stratified outcomes, Grade 1–2 adverse events showed comparatively consistent risk increases across ICI-based regimens and constituted the main contributor to the overall toxicity burden. In contrast, effect estimates for Grade 3–4 adverse events were highly uncertain (with CIs frequently spanning 1.0), indicating trend-level signals.

**Figure 5** summarizes the SUCRA values (%) for each regimen across endocrine adverse events. Higher SUCRA value corresponds to lower incidence of an endocrine adverse event. Mean_SUCRA value represents the mean SUCRA across endpoints, providing an overall comparison of the “toxicity burden” at the regimen level. Overall, conventional therapy maintained the highest Mean_SUCRA value (87.4), indicating the lowest endocrine toxicity burden. In contrast, all ICI-based regimens had lower Mean_SUCRA values, suggesting increased risk, with the following order from lower to higher risk: ICI+Chem+Antiangio-Ab (51.2), ICI+TKI (37.7), pembrolizumab (33.8), and avelumab (26.5).

Endpoint-specific patterns were more evident for thyroid-related outcomes: pembrolizumab had the lowest SUCRA value for hypothyroidism (52.5), while ICI+TKI had the lowest for hyperthyroidism (51.1). For grade-stratified outcomes, all ICI regimens showed consistently lower SUCRA values for Grade 1–2 adverse events compared to conventional therapy (e.g., ICI+TKI 49.1, pembrolizumab 40.6, avelumab 21.6), suggesting a broadly consistent increase in mild-to-moderate toxicity burden across regimens. However, ranking differences for Grade 3–4 adverse events were comparatively limited and constrained by the available information. Notably, avelumab could not be ranked (NA value) for some endpoints because the network meta-analysis included no direct or indirect comparisons involving avelumab for those outcomes. Overall, these rankings in **Figure 5** aligned with the direction of effect sizes (RRs) in **Figure 4**: conventional therapy showed the lowest overall risk, while ICI-based regimens exhibited varying degrees of risk elevation, particularly for thyroid outcomes and the overall toxicity burden.

## 4 Discussion

In this study, we conducted a systematic network meta-analysis to compare endocrine adverse event profiles between ICI monotherapy and combination regimens in CRC patients. Notably, the results indicate that ICI-based treatments overall carry a greater burden of endocrine toxicity than conventional therapies. We observed a distinct “regimen-intensity gradient” of risk, particularly pronounced for thyroid-related outcomes and overall adverse events (17, 18). From this perspective, our findings help build a more practical evidence framework, advocating a shift in CRC immunotherapy decision-making from focusing solely on benefits toward explicitly balancing efficacy with safety (18).

Biologically speaking, endocrine glands are known to be highly sensitive to disruptions in immune tolerance (19). When PD-1/PD-L1 or CTLA-4 pathways are blocked, effector T cells and autoreactive clones expand, potentially causing glandular injury via cytotoxic effects, inflammatory cytokines, and autoantibodies. These processes can manifest clinically as thyroiditis-like changes, loss of glandular reserve, or suppression of entire endocrine axes. Furthermore, certain combination strategies may compound immune activation and tissue stress. For instance, adding TKIs or anti-angiogenic agents can alter microvascular perfusion, antigen presentation, and the local cytokine environment, effectively “amplifying” immune-mediated inflammation in target organs (20, 21). From this mechanistic standpoint, a plausible explanation emerges for why combination regimens showed higher toxicity rankings than monotherapy.

Thyroid toxicity stands out as the most frequent endocrine irAE linked to ICIs. Our analysis revealed that both PD-1 monotherapy and combinations of ICI with TKIs significantly raised hypothyroidism risk compared to conventional therapy (5). Also, regimens combining ICIs with anti-angiogenic agents showed a higher risk signal in our ranking analysis, with slightly lower evidence precision. This toxicity often evolves along a continuum: an initial “transient thyrotoxicosis” phase typically gives way to secondary (and sometimes permanent) hypothyroidism. This progression mirrors the dynamic nature of immune-mediated thyroiditis, shifting from an acute inflammatory release of hormones to the gradual destruction of thyroid follicles. In CRC, as first-line immunotherapy for MSI-H/dMMR disease becomes the norm and as follow-up duration grows, thyroid dysfunction is likely to emerge as a prominent long-term safety concern (7). Although it may not directly prompt treatment discontinuation, thyroid toxicity can nonetheless worsen patients’ quality of life and compromise therapy continuity—through chronic fatigue, an elevated risk of arrhythmias, metabolic complications, and the need for long-term hormone replacement. Hence, our analysis underscores the importance of baseline TSH/FT4 screening and early on-treatment monitoring in standardized care pathways (7).

For hyperthyroidism, our analysis found a more pronounced increase in risk for certain ICI combination regimens compared to conventional therapy, while effect estimates for monotherapy were relatively imprecise. At least two factors likely contribute to this observation. First, hyperthyroidism often manifests as an early, transient phase of thyroiditis (22), which means its detection in trials heavily depends on the timing of follow-up visits and how intensively thyroid function is tested. Second, ICI combination regimens may provoke a stronger inflammatory peak, making early thyroid inflammation more likely to be captured and reported as a “hyperthyroidism/thyroiditis” event. Therefore, when interpreting hyperthyroidism outcomes, it is more appropriate to focus on the sensitivity and timing of monitoring, rather than relying solely on statistical significance from pairwise comparisons.

For thyroiditis and immune-related diabetes mellitus, although the sparse event counts across studies led to wide CIs, our ranking analysis suggest a potentially elevated risk profile for ICI combined with TKIs for both outcomes. Biologically, TKIs may increase tissue vulnerability by inducing endothelial and metabolic stress, thus lowering the threshold for clinically apparent immune-mediated injury (23). It is also important to note that although ICI-related diabetes is rare, it can emerge suddenly and may present with acute ketoacidosis. Management of such cases depends critically on early recognition—through regular monitoring of glucose and ketones—and on a coordinated, multidisciplinary approach, rather than solely on symptomatic treatment (24).

Adrenal insufficiency was another outcome with imprecise estimates in our study; however, its clinical importance should not be dismissed. Clinically, adrenal insufficiency can manifest with symptoms like fatigue, hyponatremia, hypotension, or even progress to adrenal crisis, and the risk can dramatically escalate when patients face additional stressors such as infection, surgery, or dehydration. Mechanistically, adrenal insufficiency in the ICI context could stem from primary adrenal inflammation (adrenalitis) or from immune-mediated effects on the pituitary–adrenal axis. In CRC trials specifically, routine assessment of adrenal or pituitary hormone levels is uncommon, which inherently limits detection and accurate phenotyping of these events. In practice, this implies that clinicians should keep a low threshold for screening adrenal function—typically with a morning cortisol (and ACTH when available)—in any patient with unexplained hypotension, hyponatremia, or persistent fatigue. Clinicians must also prioritize the rapid diagnosis and management of potential adrenal crisis in emergency settings (25, 26).

Our grade-specific analysis further clarified the composition of this “toxicity burden.” We observed that mild-to-moderate endocrine events were relatively common with ICI-based regimens. In contrast, severe events were overall rare. This pattern fits with clinical experience: many endocrine irAEs do not manifest as acute, high-grade toxicities but instead lead to chronic hormone deficiencies that require long-term replacement, entailing an appreciable real-world management burden (27). As such, the lack of clear differences among regimens in severe events should be viewed as evidence of insufficient statistical power rather than proof of equal risk.

Our results are consistent with previous cross-tumor safety reviews: thyroid-related outcomes remain the dominant dimension of endocrine toxicity with ICIs, and combination regimens generally raise the overall toxicity burden (28). Importantly, this study translates that consensus into the specific landscape of CRC treatment. For example, PD-1–based monotherapies and dual-immunotherapy approaches for MSI-H/dMMR CRC are rapidly becoming the standard in first- and later-line settings (15). At the same time, immunotherapy combinations for pMMR/MSS CRC—such as PD-L1 inhibitors plus bevacizumab or chemotherapy, or PD-1 inhibitors plus TKIs—are still under active investigation (29). In both cases, it is essential to quantify any marginal efficacy gains along with the safety trade-offs. A key contribution of our study is the use of a network meta-analytic framework to integrate both direct and indirect evidence, generating comparative rankings of multiple regimens on a unified scale. This yields more structured evidence to guide regimen selection and set priorities for endocrine adverse event monitoring.

There are several limitations in this study. First, our outcome-specific networks did not form closed loops, so we could not perform formal global or local inconsistency checks, including formal consistency testing between direct and indirect comparisons. Consequently, our ranking results rely heavily on indirect comparisons within star-shaped networks and on the assumption of a common comparator. This methodological limitation means that subtle differences between regimens should be interpreted with caution—focusing on risk stratification rather than asserting precise effect sizes. Second, the analysis was based on a very limited number of RCTs (n=6 overall), with several outcomes informed by data from only three studies. The combination of the small number of included studies and the low event rates resulted in wide 95% CIs and limited statistical power. This is particularly problematic for the calculation of SUCRA rankings, which are highly sensitive to imprecision in effect estimates; thus, all ranking results should be interpreted as exploratory and hypothesis-generating, rather than definitive, due to these statistical constraints. Third, substantial clinical heterogeneity existed across the included trials, including differences in molecular subtype, treatment line, and therapeutic combinations. These differences are not merely potential effect modifiers, but are likely to directly influence the incidence of endocrine adverse events, as tumor biology and co-administered therapies are both established modulators of immunotherapy-related adverse effects. Fourth, variations in monitoring schedules for endocrine function and standards for adverse event reporting across trials may introduce detection bias, particularly for transient events such as thyroid dysfunction, where mild or subclinical cases may be under-ascertained in trials with less intensive testing protocols. These inconsistencies in outcome ascertainment are a potential source of heterogeneity in our aggregate trial-level analysis. Additionally, our analysis used aggregate trial-level data, which means we could not adjust for key individual patient factors such as baseline endocrine status, autoantibodies, prior treatments, or comorbidities, all of which are known to influence the risk of endocrine adverse events. Future studies—such as prospective cohorts or individual patient data meta-analyses—should validate the robustness of these rankings under more uniform testing and reporting conditions and explore clinically actionable predictors (e.g., baseline thyroid autoantibodies, specific inflammatory signatures, or HLA profiles). Larger studies with extended follow-up and more uniform adverse event reporting are needed to confirm the robustness of these rankings, to identify high-risk patient subgroups and modifiable predictors, and thereby move CRC immunotherapy toward a more sustainable, manageable paradigm.

## 5 Conclusion

This network meta-analysis of RCTs in CRC indicates that, compared with conventional therapy, ICI-based regimens overall increase the burden of endocrine adverse events, with thyroid-related toxicity emerging as the most consistent and clinically actionable safety signal. In clinical practice, endocrine adverse event monitoring should be initiated early and standardized—particularly baseline thyroid function assessment and dynamic surveillance during the early treatment phase. Furthermore, the apparent gradient of risk among combination strategies implies that real-world CRC immunotherapy decisions should explicitly balance efficacy against potential toxicity risks to support sustained treatment continuity.
