# Supplementary material for: A network meta-analysis of endocrine adverse events induced by immune checkpoint inhibitors in colorectal cancer
Source: Front Immunol. 2026 Jul 10;17:1798732. doi: 10.3389/fimmu.2026.1798732 (PMC13395773; doi:10.3389/fimmu.2026.1798732)
Supplement: Supplementary file 1 [file DataSheet1.docx]

**Supplement Materials**

A network meta-analysis of endocrine adverse events induced by immune checkpoint inhibitors in colorectal cancer.

|  | Pages |
| --- | --- |
| Supplement S1: PRISMA 2020 checklist to include when reporting a systematic review involving a network meta-analysis. | 2-5 |
| Supplement S2: The searching strategy and results from electronic databases | 6-8 |
| Supplement S3: Supplementary Figures S1–S7 | 9-15 |
| Supplementary Table S4: Full-text reports excluded, with reasons | 16-18 |

Supplement S1: PRISMA 2020 checklist to include when reporting a systematic review involving a network meta-analysis.


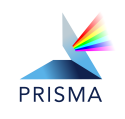
 **PRISMA 2020 Checklist**

| **Section and Topic** | **Item #** | **Checklist item** | **Location where item is reported** |
| --- | --- | --- | --- |
| **TITLE** | | |  |
| Title | 1 | Identify the report as a systematic review. | 1 |
| **ABSTRACT** | | |  |
| Abstract | 2 | See the PRISMA 2020 for Abstracts checklist. | 1-2 |
| **INTRODUCTION** | | |  |
| Rationale | 3 | Describe the rationale for the review in the context of existing knowledge. | 2-3 |
| Objectives | 4 | Provide an explicit statement of the objective(s) or question(s) the review addresses. | 3-4 |
| **METHODS** | | |  |
| Eligibility criteria | 5 | Specify the inclusion and exclusion criteria for the review and how studies were grouped for the syntheses. | 4-5 |
| Information sources | 6 | Specify all databases, registers, websites, organisations, reference lists and other sources searched or consulted to identify studies. Specify the date when each source was last searched or consulted. | 4 |
| Search strategy | 7 | Present the full search strategies for all databases, registers and websites, including any filters and limits used. | 4 |
| Selection process | 8 | Specify the methods used to decide whether a study met the inclusion criteria of the review, including how many reviewers screened each record and each report retrieved, whether they worked independently, and if applicable, details of automation tools used in the process. | 4 |
| Data collection process | 9 | Specify the methods used to collect data from reports, including how many reviewers collected data from each report, whether they worked independently, any processes for obtaining or confirming data from study investigators, and if applicable, details of automation tools used in the process. | 5 |
| Data items | 10a | List and define all outcomes for which data were sought. Specify whether all results that were compatible with each outcome domain in each study were sought (e.g. for all measures, time points, analyses), and if not, the methods used to decide which results to collect. | 5 |
|  | 10b | List and define all other variables for which data were sought (e.g. participant and intervention characteristics, funding sources). Describe any assumptions made about any missing or unclear information. | 5 |
| Study risk of bias assessment | 11 | Specify the methods used to assess risk of bias in the included studies, including details of the tool(s) used, how many reviewers assessed each study and whether they worked independently, and if applicable, details of automation tools used in the process. | 5-6 |
| Effect measures | 12 | Specify for each outcome the effect measure(s) (e.g. risk ratio, mean difference) used in the synthesis or presentation of results. | 6 |
| Synthesis methods | 13a | Describe the processes used to decide which studies were eligible for each synthesis (e.g. tabulating the study intervention characteristics and comparing against the planned groups for each synthesis (item #5)). | 6-7 |
|  | 13b | Describe any methods required to prepare the data for presentation or synthesis, such as handling of missing summary statistics, or data conversions. | 6 |
|  | 13c | Describe any methods used to tabulate or visually display results of individual studies and syntheses. | 6 |
|  | 13d | Describe any methods used to synthesize results and provide a rationale for the choice(s). If meta-analysis was performed, describe the model(s), method(s) to identify the presence and extent of statistical heterogeneity, and software package(s) used. | 6 |
|  | 13e | Describe any methods used to explore possible causes of heterogeneity among study results (e.g. subgroup analysis, meta-regression). | 6 |
|  | 13f | Describe any sensitivity analyses conducted to assess robustness of the synthesized results. | NR |
| Reporting bias assessment | 14 | Describe any methods used to assess risk of bias due to missing results in a synthesis (arising from reporting biases). | 6 |
| Certainty assessment | 15 | Describe any methods used to assess certainty (or confidence) in the body of evidence for an outcome. | NR |
| **RESULTS** | | |  |
| Study selection | 16a | Describe the results of the search and selection process, from the number of records identified in the search to the number of studies included in the review, ideally using a flow diagram. | 6 |
|  | 16b | Cite studies that might appear to meet the inclusion criteria, but which were excluded, and explain why they were excluded. | 6 |
| Study characteristics | 17 | Cite each included study and present its characteristics. | 6 |
| Risk of bias in studies | 18 | Present assessments of risk of bias for each included study. | 7 |
| Results of individual studies | 19 | For all outcomes, present, for each study: (a) summary statistics for each group (where appropriate) and (b) an effect estimate and its precision (e.g. confidence/credible interval), ideally using structured tables or plots. | NR |
| Results of syntheses | 20a | For each synthesis, briefly summarise the characteristics and risk of bias among contributing studies. | 7-9 |
|  | 20b | Present results of all statistical syntheses conducted. If meta-analysis was done, present for each the summary estimate and its precision (e.g. confidence/credible interval) and measures of statistical heterogeneity. If comparing groups, describe the direction of the effect. | 8-9 |
|  | 20c | Present results of all investigations of possible causes of heterogeneity among study results. | NR |
|  | 20d | Present results of all sensitivity analyses conducted to assess the robustness of the synthesized results. | NR |
| Reporting biases | 21 | Present assessments of risk of bias due to missing results (arising from reporting biases) for each synthesis assessed. | NR |
| Certainty of evidence | 22 | Present assessments of certainty (or confidence) in the body of evidence for each outcome assessed. | NR |
| **DISCUSSION** | | |  |
| Discussion | 23a | Provide a general interpretation of the results in the context of other evidence. | 9 |
|  | 23b | Discuss any limitations of the evidence included in the review. | 12 |
|  | 23c | Discuss any limitations of the review processes used. | 12-13 |
|  | 23d | Discuss implications of the results for practice, policy, and future research. | 12-13 |
| **OTHER INFORMATION** | | |  |
| Registration and protocol | 24a | Provide registration information for the review, including register name and registration number, or state that the review was not registered. | 4 |
|  | 24b | Indicate where the review protocol can be accessed, or state that a protocol was not prepared. | NR |
|  | 24c | Describe and explain any amendments to information provided at registration or in the protocol. | NR |
| Support | 25 | Describe sources of financial or non-financial support for the review, and the role of the funders or sponsors in the review. | 13 |
| Competing interests | 26 | Declare any competing interests of review authors. | 13 |
| Availability of data, code and other materials | 27 | Report which of the following are publicly available and where they can be found: template data collection forms; data extracted from included studies; data used for all analyses; analytic code; any other materials used in the review. | 13 |

Supplement S2: The searching strategy and results from electronic databases

| **Search** | **Query** | **Items found** | |
| --- | --- | --- | --- |
| **Pubmed (the retrieval time: 20251121)** | | | |
| #1 | "Immune Checkpoint Inhibitors"[Mesh] OR "Programmed Cell Death 1 Receptor"[Mesh] OR "Programmed Cell Death 1 Ligand 1 Protein"[Mesh] OR "CTLA-4 Antigen"[Mesh] OR "immune checkpoint inhibitor*"[tiab] OR "immune checkpoint blockade"[tiab] OR "immune checkpoint inhibition"[tiab] OR "PD-1"[tiab] OR "PD-L1"[tiab] OR "CTLA-4"[tiab] | 91,355 | |
| #2 | "Colorectal Neoplasms"[Mesh] OR "Gastrointestinal Neoplasms"[Mesh] OR "colorectal"[tiab] OR "colorectal cancer"[tiab] OR "colorectal carcinoma"[tiab] OR "CRC"[tiab] OR "colon"[tiab] OR "rectal"[tiab] OR "gastrointestinal cancer*"[tiab] OR "gastrointestinal neoplasm*"[tiab] OR "cancer of the gastrointestinal tract"[tiab] OR "gastric"[tiab] OR "stomach"[tiab] | 384,109 | |
| #3 | "irAE*"[tiab] OR "immune-related adverse event*"[tiab] OR "immune-mediated adverse event*"[tiab] OR "treatment-related adverse event*"[tiab] OR "treatment-related AE*"[tiab] OR "select adverse event*"[tiab] OR "select AE*"[tiab] OR "select treatment-related adverse event*"[tiab] OR "select treatment-related AE*"[tiab] | 15983 | |
| #4 | #1 AND #2 AND #3 | 772 | |
| **Cochrane Library (the retrieval time: 20251121)** | | |  |
| #1 | Immune Checkpoint Inhibitors | 2078 |  |
| #2 | Programmed Cell Death 1 Receptor | 733 |  |
| #3 | Programmed Cell Death 1 Ligand 1 Protein | 487 |  |
| #4 | CTLA-4 Antigen | 289 |  |
| #5 | "immune checkpoint inhibitor*" OR "immune checkpoint blockade" OR "immune checkpoint inhibition" OR "PD-1" OR "PD-L1" OR "CTLA-4 | 1,280,907 |  |
| #6 | #1 OR #2 OR #3 OR #4 OR #5 | 1,280,932 |  |
| #7 | Colorectal Neoplasms | 11,811 |  |
| #8 | Gastrointestinal Neoplasms | 6,599 |  |
| #9 | colorectal OR "colorectal cancer" OR "colorectal carcinoma" OR CRC OR colon OR rectal OR (gastrointestinal NEXT cancer*) OR (gastrointestinal NEXT neoplasm*) OR "cancer of the gastrointestinal tract" OR gastric OR stomach | 97,593 |  |
| #10 | #7 OR #8 OR #9 | 100,364 |  |
| #11 | irAE* OR (immune-related NEXT adverse NEXT event*) OR (immune-mediated NEXT adverse NEXT event*) OR (treatment-related NEXT adverse NEXT event*) OR (treatment-related NEXT AE*) OR (select NEXT adverse NEXT event*) OR (select NEXT AE*) OR (select NEXT treatment-related NEXT adverse NEXT event*) OR (select NEXT treatment-related NEXT AE*) | 6,890 |  |
| #12 | #6 AND #10 AND #11 | 585 |  |
| **Embase (the retrieval time: 20251122)** | | |  |
| #1 | 'immune checkpoint inhibitor'/exp OR 'programmed cell death 1 receptor'/exp OR 'programmed cell death 1 ligand 1'/exp OR 'ctla 4 antigen'/exp OR 'immune checkpoint inhibitor*':ti,ab,kw OR 'immune checkpoint blockade':ti,ab,kw OR 'immune checkpoint inhibition':ti,ab,kw OR 'pd-1':ti,ab,kw OR 'pd1':ti,ab,kw OR 'pd-l1':ti,ab,kw OR 'pdl1':ti,ab,kw OR 'ctla-4':ti,ab,kw OR pembrolizumab:ti,ab,kw OR nivolumab:ti,ab,kw OR ipilimumab:ti,ab,kw OR tremelimumab:ti,ab,kw OR cemiplimab:ti,ab,kw OR sintilimab:ti,ab,kw OR toripalimab:ti,ab,kw OR tislelizumab:ti,ab,kw OR camrelizumab:ti,ab,kw OR atezolizumab:ti,ab,kw OR durvalumab:ti,ab,kw OR avelumab:ti,ab,kw | 272,975 |  |
| #2 | 'colorectal neoplasm'/exp OR 'gastrointestinal neoplasm' OR colorectal:ti,ab,kw OR 'colorectal cancer':ti,ab,kw OR 'colorectal carcinoma':ti,ab,kw OR 'colorectal tumor*':ti,ab,kw OR 'colorectal tumour*':ti,ab,kw OR crc:ti,ab,kw OR colon:ti,ab,kw OR rectal:ti,ab,kw OR 'colon cancer':ti,ab,kw OR 'colon carcinoma':ti,ab,kw OR 'rectal cancer':ti,ab,kw OR 'rectal carcinoma':ti,ab,kw OR 'gastrointestinal cancer*':ti,ab,kw OR 'gastrointestinal neoplasm*':ti,ab,kw OR 'gastrointestinal carcinoma*':ti,ab,kw OR 'gastrointestinal tumor*':ti,ab,kw OR 'gastrointestinal tumour*':ti,ab,kw OR 'cancer of the gastrointestinal tract':ti,ab,kw OR 'gastric cancer':ti,ab,kw OR 'gastric carcinoma':ti,ab,kw OR 'stomach cancer':ti,ab,kw OR 'stomach carcinoma':ti,ab,kw | 1,062,484 |  |
| #3 | irae*:ti,ab,kw OR 'immune-related adverse event*':ti,ab,kw OR 'immune related adverse event*':ti,ab,kw OR 'immune-mediated adverse event*':ti,ab,kw OR 'immune mediated adverse event*':ti,ab,kw OR 'treatment-related adverse event*':ti,ab,kw OR 'treatment related adverse event*':ti,ab,kw OR 'treatment-related ae*':ti,ab,kw OR 'treatment related ae*':ti,ab,kw OR 'select adverse event*':ti,ab,kw OR 'select ae*':ti,ab,kw OR 'select treatment-related adverse event*':ti,ab,kw OR 'select treatment related adverse event*':ti,ab,kw OR 'select treatment-related ae*':ti,ab,kw OR 'select treatment related ae*':ti,ab,kw OR 'immune-related toxicity':ti,ab,kw OR 'immune mediated toxicity':ti,ab,kw OR 'immunotherapy-related toxicity':ti,ab,kw OR 'immunotherapy related toxicity':ti,ab,kw OR endocrinopath*:ti,ab,kw OR 'endocrine adverse event*':ti,ab,kw OR 'endocrine toxicity':ti,ab,kw OR 'endocrine dysfunction*':ti,ab,kw OR 'endocrine disorder*':ti,ab,kw OR hypothyroidism:ti,ab,kw OR hyperthyroidism:ti,ab,kw OR thyroiditis:ti,ab,kw OR 'thyroid dysfunction*':ti,ab,kw OR 'thyroid disorder*':ti,ab,kw OR hypophysitis:ti,ab,kw OR 'adrenal insufficiency':ti,ab,kw OR 'diabetes mellitus':ti,ab,kw | 674,281 |  |
| #4 | #1 AND #2 AND #3 | 2,427 |  |
| #5 | #4 AND [2015-2025]/py | 2,168 |  |
| #6 | #5 AND [humans]/lim | 2,146 |  |
| #7 | #6 AND [english]/lim | 2,079 |  |

Supplement S3: Supplementary Figures

**Hypothyroidism**

The league table (Supplementary Figure S1A) showed that, compared with conventional therapy, pembrolizumab significantly increased the risk of hypothyroidism (RR = 4.44, 95% CI 1.55–12.74), and ICI plus a tyrosine kinase inhibitor (ICI+TKI) also significantly increased this risk (RR = 5.62, 95% CI 3.41–9.26). The differences were not statistically significant for ICI+chemotherapy+anti-angiogenic antibody (ICI+Chemo+Antiangio-Ab) versus conventional therapy (RR = 7.82, 95% CI 0.95–64.26) or for avelumab versus conventional therapy (RR = 13.20, 95% CI 0.76–229.55). Apart from these comparisons, no other pairwise differences between regimens were statistically significant (all 95% CIs crossed 1). The SUCRA rankings (Supplementary Figure S1B) indicated that the risk of hypothyroidism increased (SUCRA decreased) in the following order: conventional therapy (98.2%, MeanRank = 1.1), pembrolizumab (52.5%, 2.9), ICI+TKI (42.1%, 3.3), ICI+Chemo+Antiangio-Ab (33.8%, 3.6), and avelumab (23.4%, 4.1).

A


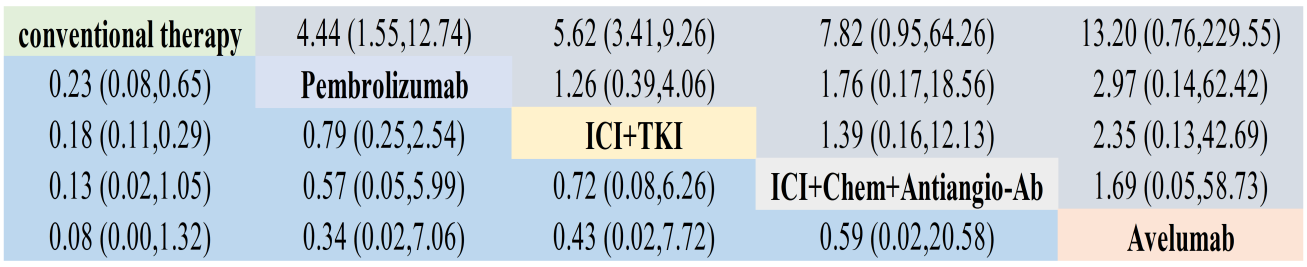


B


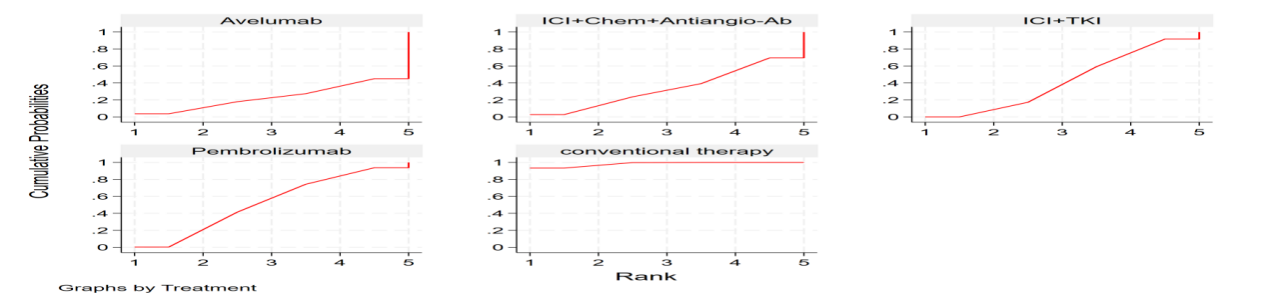


Supplementary Figure S1. League table (A) and ranking probability plot (B) for hypothyroidism.

**Hyperthyroidism**

As shown in the league table (Supplementary Figure S2A), compared with conventional therapy, ICI plus a tyrosine kinase inhibitor (ICI+TKI) significantly increased the risk of hyperthyroidism (RR = 5.43, 95% CI 1.22–24.24), and ICI plus chemotherapy plus an anti-angiogenic antibody (ICI+Chemo+Antiangio-Ab) also significantly increased this risk (RR = 9.86, 95% CI 1.29–75.58). Pembrolizumab (RR = 12.16, 95% CI 0.69–213.85) and avelumab (RR = 11.17, 95% CI 0.63–197.91) did not differ significantly from conventional therapy. Apart from these comparisons, no other pairwise differences between regimens were statistically significant (all 95% CIs crossed 1). The SUCRA rankings (Supplementary Figure S2B) suggested that the risk of hyperthyroidism increased (SUCRA decreased) in the following order: conventional therapy (97.0%, MeanRank = 1.1), ICI+TKI (51.1%, 3.0), ICI+Chemo+Antiangio-Ab (35.1%, 3.6), avelumab (34.4%, 3.6), and pembrolizumab (32.3%, 3.7).

A


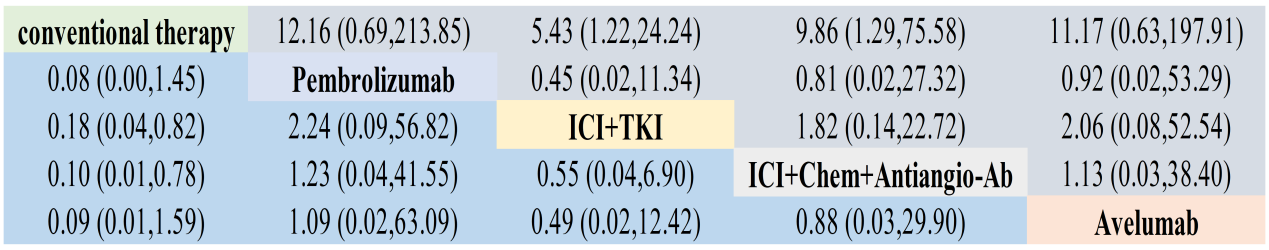


B


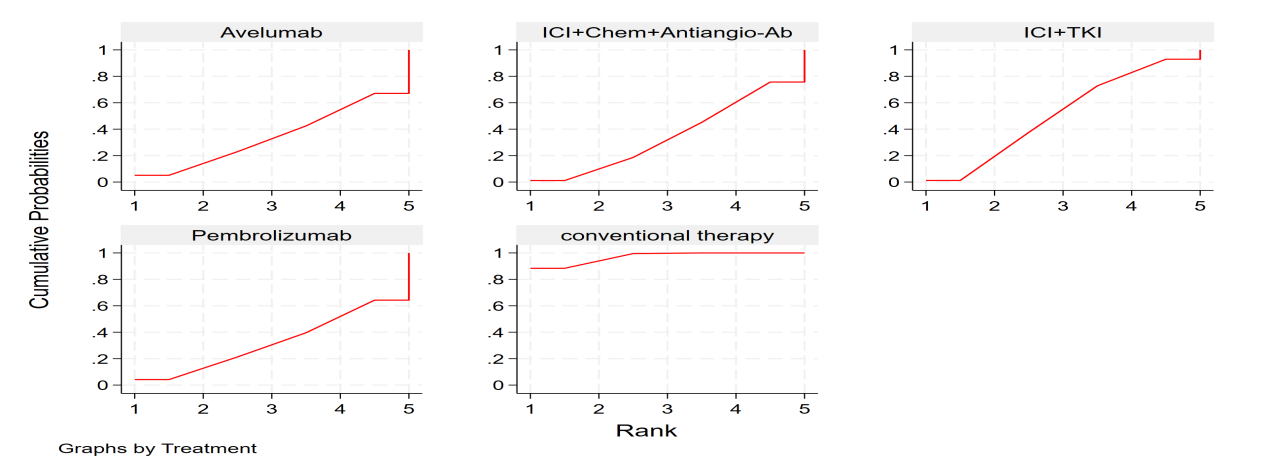


Supplementary Figure S2. League table (A) and ranking probability plot (B) for hyperthyroidism.

**Adrenal insufficiency**

As shown in the league table (Supplementary Figure S3A), no statistically significant differences were observed between conventional therapy and pembrolizumab (RR = 12.16, 95% CI 0.69–213.85), ICI plus a tyrosine kinase inhibitor (ICI+TKI) (RR = 3.95, 95% CI 0.44–35.08), or ICI plus chemotherapy plus an anti-angiogenic antibody (ICI+Chemo+Antiangio-Ab) (RR = 1.53, 95% CI 0.26–8.96), as all 95% CIs crossed 1. Apart from these comparisons, no other pairwise differences between regimens were statistically significant (all 95% CIs crossed 1), indicating overall imprecision in the estimates for this outcome. The SUCRA rankings (Supplementary Figure S3B) indicated that the risk of adrenal insufficiency increased (SUCRA decreased) in the following order: conventional therapy (84.5%, MeanRank = 1.5), ICI+Chemo+Antiangio-Ab (65.1%, 2.0), ICI+TKI (36.2%, 2.9), and pembrolizumab (14.2%, 3.6).

A


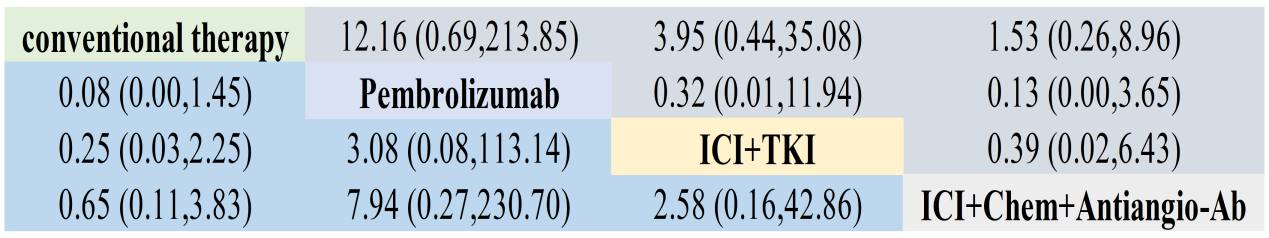


B


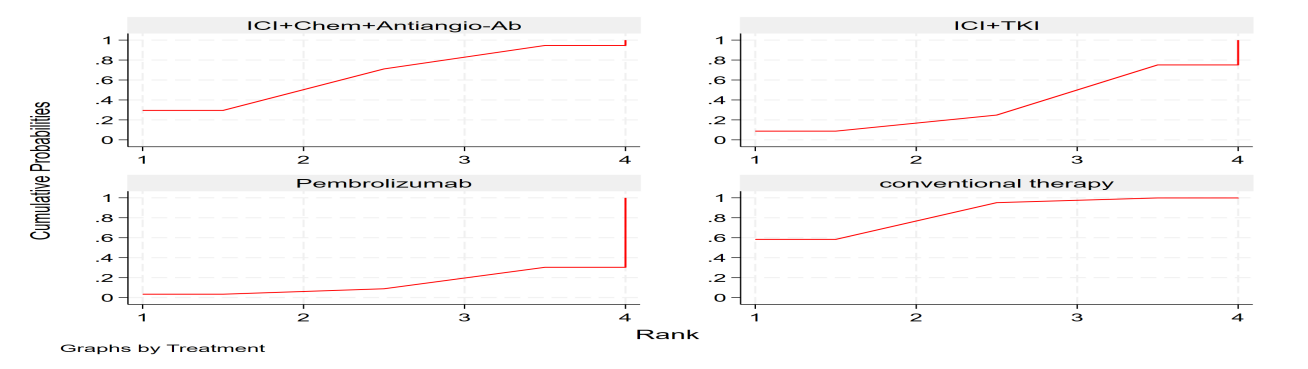


Supplementary Figure S3.League table (A) and ranking probability plot (B) for adrenal insufficiency.

**Thyroiditis**

As shown in the league table (Supplementary Figure S4A), compared with conventional therapy, no statistically significant differences were observed for pembrolizumab (RR = 4.68, 95% CI 0.23–96.56), ICI plus a tyrosine kinase inhibitor (ICI+TKI) (RR = 10.86, 95% CI 0.60–195.34), or ICI plus chemotherapy plus an anti-angiogenic antibody (ICI+Chemo+Antiangio-Ab) (RR = 1.47, 95% CI 0.06–35.85), as all 95% CIs crossed 1. Apart from these comparisons, no other pairwise differences between regimens were statistically significant (all 95% CIs crossed 1). The SUCRA rankings (Supplementary Figure S4B) indicated that the risk of thyroiditis increased (SUCRA decreased) in the following order: conventional therapy (80.0%, MeanRank = 1.6), ICI+Chemo+Antiangio-Ab (63.6%, 2.1), pembrolizumab (36.6%, 2.9), and ICI+TKI (19.8%, 3.4).

A


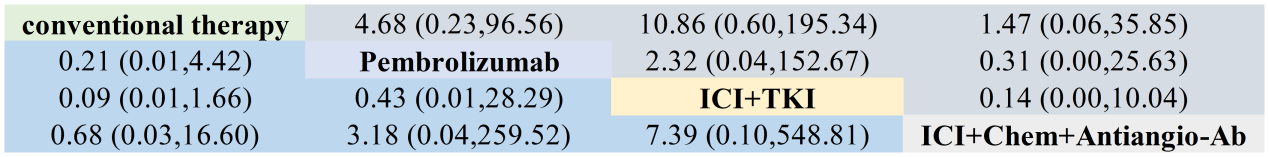


B


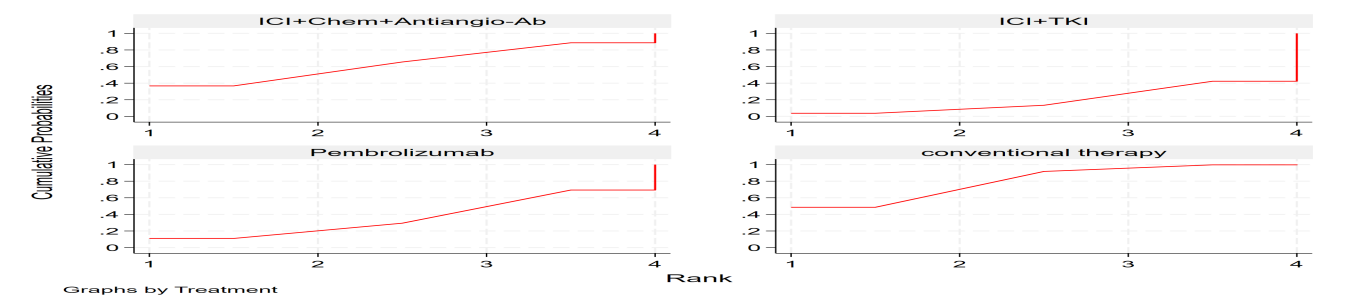


Supplementary Figure S4.League table (A) and ranking probability plot (B) for thyroiditis.

**Diabetes mellitus**

As shown in the league table (Supplementary Figure S5A), compared with conventional therapy, no statistically significant differences were observed for pembrolizumab (RR = 4.68, 95% CI 0.23–96.56), ICI plus a tyrosine kinase inhibitor (ICI+TKI) (RR = 4.94, 95% CI 0.24–102.29), or ICI plus chemotherapy plus an anti-angiogenic antibody (ICI+Chemo+Antiangio-Ab) (RR = 1.62, 95% CI 0.07–39.01), as all 95% CIs crossed 1. Apart from these comparisons, no other pairwise differences between regimens were statistically significant (all 95% CIs crossed 1). The SUCRA rankings (Supplementary Figure S5B) indicated that the risk of diabetes mellitus increased (SUCRA decreased) in the following order: conventional therapy (76.9%, MeanRank = 1.7), ICI+Chemo+Antiangio-Ab (58.0%, 2.3), pembrolizumab (33.1%, 3.0), and ICI+TKI (32.0%, 3.0).

A


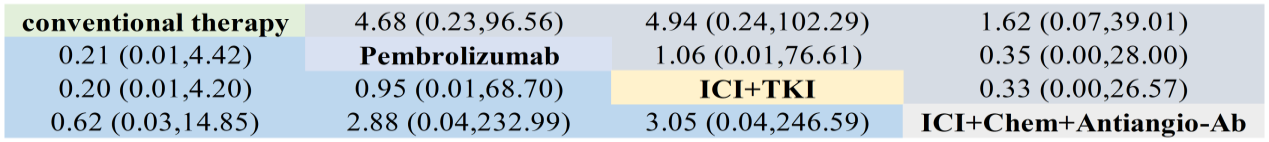


B


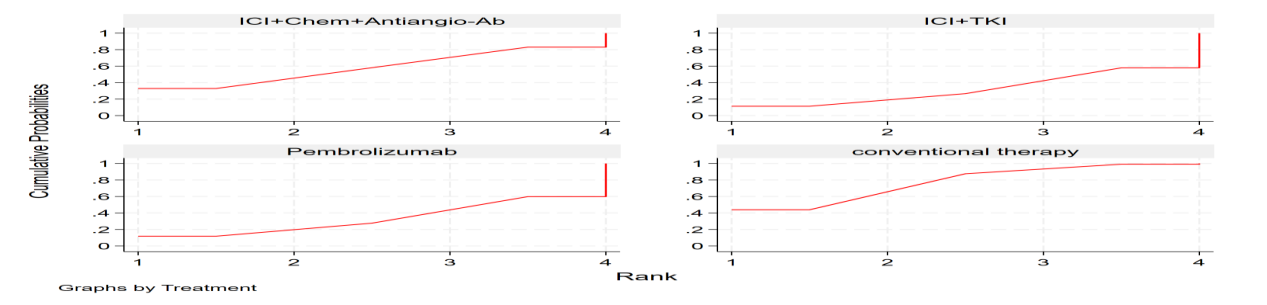


Supplementary Figure S5.League table (A) and ranking probability plot (B) for diabetes mellitus.

**Grade 1–2 adverse events**

As shown in the league table (Supplementary Figure S6A), compared with conventional therapy, pembrolizumab significantly increased the risk of grade 1–2 adverse events (RR = 7.94, 95% CI 2.89–21.83). ICI plus a tyrosine kinase inhibitor (ICI+TKI) also significantly increased this risk (RR = 5.77, 95% CI 3.67–9.06), and ICI plus chemotherapy plus an anti-angiogenic antibody (ICI+Chemo+Antiangio-Ab) likewise significantly increased the risk (RR = 5.89, 95% CI 1.52–22.77). Avelumab showed the most pronounced increase in risk (RR = 23.36, 95% CI 1.41–388.12). Apart from comparisons versus conventional therapy, no statistically significant differences were observed among the ICI-based regimens (all 95% CIs crossed 1). The SUCRA rankings (Supplementary Figure S6B) indicated that the risk of grade 1–2 adverse events increased (SUCRA decreased) in the following order: conventional therapy (96.4%, MeanRank = 1.1), ICI+TKI (49.1%, 3.0), ICI+Chemo+Antiangio-Ab (42.2%, 3.3), pembrolizumab (40.6%, 3.4), and avelumab (21.6%, 4.1).

A


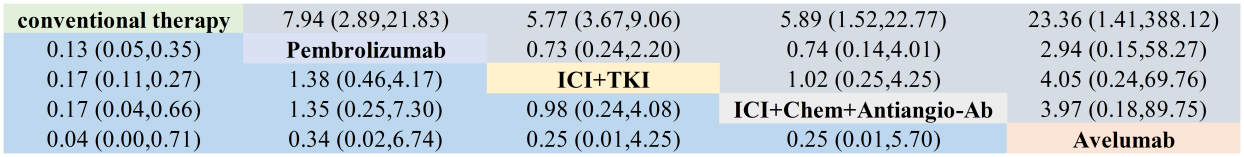


B


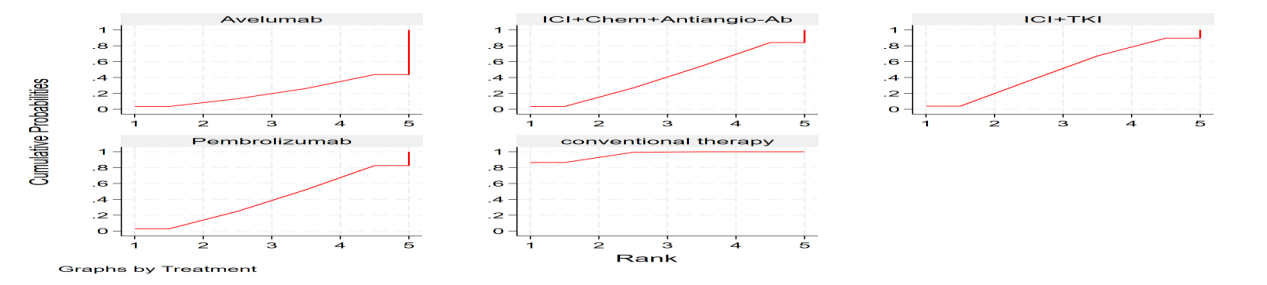


Supplementary Figure S6.League table (A) and ranking probability plot (B) for grade 1–2 adverse events.

**Grade 3–4 adverse events**

As shown in the league table (Supplementary Figure S7A), compared with conventional therapy, no statistically significant differences were observed for pembrolizumab (RR = 6.55, 95% CI 0.34–125.62), ICI plus a tyrosine kinase inhibitor (ICI+TKI) (RR = 4.94, 95% CI 0.24–102.29), or ICI plus chemotherapy plus an anti-angiogenic antibody (ICI+Chemo+Antiangio-Ab) (RR = 1.62, 95% CI 0.07–39.01), as all 95% CIs crossed 1. No statistically significant differences were observed in other pairwise comparisons between regimens (all 95% CIs crossed 1). The SUCRA rankings (Supplementary Figure S7B) indicated that the risk of grade 3–4 adverse events increased (SUCRA decreased) in the following order: conventional therapy (78.9%, MeanRank = 1.6), ICI+Chemo+Antiangio-Ab (60.3%, 2.2), ICI+TKI (33.3%, 3.0), and pembrolizumab (27.5%, 3.2).

A


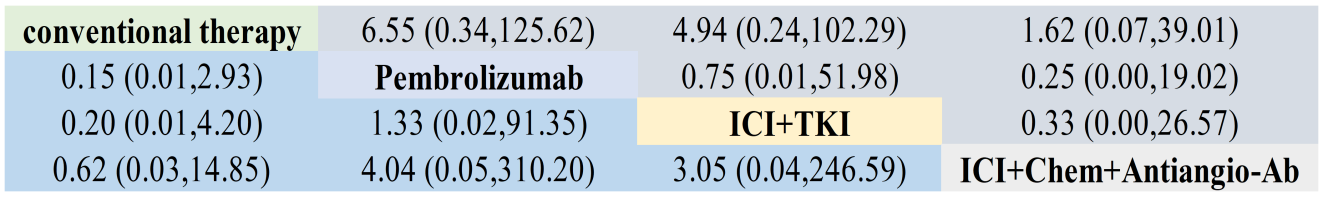


B


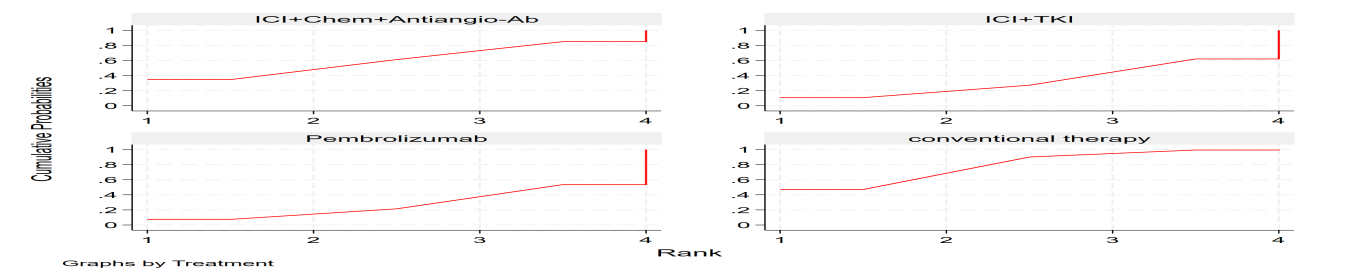


Supplementary Figure S7. League table (A) and ranking probability plot (B) for grade 3–4 adverse events.

Supplementary Table S4: Full-text reports excluded, with reasons

| **Study (First author, year)** | **Trial ID** | **Primary reason code** |
| --- | --- | --- |
| 1. Lin Z, 2021（单臂II期：SCRT+化疗+camrelizumab，LARC） | NCT04231552 | R1 |
| 2. Wang F, 2021（regorafenib+toripalimab，mCRC） | NCT02915432 | R1 |
| 3.Haag GM, 2022（pembrolizumab+maraviroc，MSS/MMRp mCRC） | NCT03274804 | R1 |
| 4.Rao S, 2022（retifanlimab，Squamous cell carcinoma of the anal canal） | NCT03597295 | R2 |
| 5. Cercek A, 2022（dostarlimab，dMMR LARC） | NCT04165772 | R1 |
| 6.Rahma OE, 2022（ziv-aflibercept+pembrolizumab，solid tumor） | NCT02298959 | R2 |
| 7. Marabelle A, 2022（KEYNOTE-158：pembrolizumab，Squamous cell carcinoma of the anal canal） | NCT02628067 | R2 |
| 8. Johnson B, 2022（durvalumab+trametinib，MSS mCRC） | NCT03428126 | R1 |
| 9. Oh CR, 2022（durvalumab ，MSI-H/dMMR mCRC） | NCT03435107 | R1 |
| 10.Yaqi Wang, 2022（TORCH：SCRT+CAPOX+toripalimab，LARC） | NCT04518280 | R4 |
| 11. Saunders MP, 2022（CXD101+nivolumab，MSS mCRC） | EudraCT: 2017-004509-42 | R1 |
| 12.Kuang C, 2022（pembrolizumab+azacitidine，mCRC） | NCT02260440 | R1 |
| 13.Levy A, 2024（SABR-PDL1：SBRT+atezolizumab；advanced pretreated CRC） | NCT02992912 | R1 |
| 14. Wang Y, 2025（sintilimab + bevacizumab + CAPOX， RAS-mut/MSS mCRC） | NCT06206096 | R1 |
| 15. Yu JH, 2024（camrelizumab + apatinib， LA MSI-H/dMMR CRC） | NCT04715633 | R1 |
| 16. Yang Z, 2024（PD-1 + Long-term radiotherapy and chemotherapy，LARC） | NCT04911517 | R1 |
| 17. Xu X, 2024（tislelizumab + cetuximab + irinotecan， MSS/RAS WT mCRC） | NCT05143099 | R1 |
| 18. Xia F, 2024（TORCH：iTNT，SCRT/CAPOX + toripalimab，pMMR/MSS LARC） | NCT04518280 | R3 |
| 19. Saeed A, 2024（CAMILLA CRC cohort：cabozantinib + durvalumab，CRC） | NCT03539822 | R1 |
| 20. Qiu MZ, 2024（SHR-1701 + XELOX + bev，mCRC） | NCT04856787 | R1 |
| 21. Overman MJ, 2024（CheckMate 142：nivolumab + relatlimab，MSI-H/dMMR mCRC） | NCT02060188 | R1 |
| 22. Levy A, 2024（SBRT + atezolizumab，CRC） | NCT02992912 | R1 |
| 23. Lentz RW, 2024（pembrolizumab + binimetinib + bevacizumab，MSS mCRC） | NCT03475004 | R3 |
| 24. Cartwright E, 2024（EMERGE：domatinostat+avelumab，MMRp OGA/CRC） | NCT03812796 | R1 |
| 25.Bessudo A, 2024（vicriviroc+pembrolizumab，MSS/pMMR mCRC） | NCT03631407 | R3 |
| 26. de Gooyer PGM, 2024（NICHE-3：nivolumab+relatlimab， dMMR ） | NCT03026140 | R1 |
| 27.Wang Y, 2025（sintilimab+bevacizumab+CAPOX；RAS-mut MSS mCRC） | NCT06206096 | R1 |
| 28. Yu JH, 2024（NEOCAP：camrelizumab+apatinib；MSI-H/dMMR CRC） | NCT04715633 | R1 |
| 29.Yang Z, 2024（PD-1 blockade+ Long-term radiotherapy and chemotherapy；MSS/pMMR LARC） | NCT04911517 | R1 |
| 30.Xu X, 2024（tislelizumab+cetuximab+irinotecan；MSS，RAS WT mCRC） | NCT05143099 | R1 |
| 31.Lentz RW, 2024（pembrolizumab+binimetinib+bevacizumab；refractory MSS mCRC） | NCT03475004 | R3 |
| 32.Saeed A,202（cabozantinib+durvalumab，chemo-refractory CRC） | NCT03539822 | R1 |
| 33.Qiu MZ, 2024（SHR-1701+XELOX+bevacizumab；unresectable mCRC） | NCT04856787 | R1 |
| 34.Overman MJ, 2024（nivolumab+relatlimab；MSI-H/dMMR mCRC） | NCT02060188 | R1 |

**Note:R1:** Not randomized controlled trial (e.g., observational, single-arm, retrospective, non-randomized).**R2:** Wrong population (not colorectal cancer, or CRC data not separable).

**R3:** Not the prespecified treatment node/regimen (cannot be mapped to the NMA network). **R4:** Conference abstract/protocol only, no full text or insufficient data after attempts.
